# Fractionation of rare earth elements in tropical soils from marine mudstone along a toposequence

**DOI:** 10.1016/j.heliyon.2025.e42097

**Published:** 2025-01-18

**Authors:** Marvin D. Cascante, Cho-Yin Wu, Zeng-Yei Hseu

**Affiliations:** Department of Agricultural Chemistry, National Taiwan University, Taipei, 10617, Taiwan

**Keywords:** Biogeochemistry, Parent material, Pedogenesis, Soil classification

## Abstract

Marine mudstones are major reservoirs for rare earth elements (REEs), and their topographical positions influence the distribution of soil REEs. This study investigated the influence of pedogenic factors on the concentration, spatial distribution, and fractionation of REEs in the soils from marine mudstone along a toposequence in Taiwan. Soil samples from four pedons were collected along the toposequence and were further analyzed for general properties and elemental composition. The results showed the highest REE concentrations (ranging from 176 to 221 mg kg^−1^) in the foot slope, while the summit had the lowest content (ranging from 95.7 to 156 mg kg^−1^), revealing the influence of the landscape on the distribution of REEs. Increasing ∑LREEs/∑HREEs ratios (ranged from 1.5 to 3.9), δCe (ranged from 0.3 to 1.0), and δEu (ranged from 1.1 to 1.4) values were observed relative to the decreasing elevation, which was dictated by the solubility and mobility of the elements during the transportation processes, provoking the fractionation of REEs. δCe positively and significantly (*p* < 0.001) correlated with dithionite-citrate-carbonate extractable iron, indicating the close interactions of iron oxides and the redox chemistry of Ce. The clustering of the samples in the principal components analysis was not observed in the distinguished prevalence of total REEs, LREEs, HREEs, and REEs fractionations, demonstrating the indirect control of the general properties to the behavior of REEs in the soils derived from marine sediments.

## 1 Introduction

The occurrence of marine sediments from the oceanic lithosphere to the continental margins is primarily generated by tectonic movement. Marine sediments are major geochemical reservoirs because their ecosystems are convergent sites of waters and sediments from riverine fluxes and the marine environment, and the deposits are not easily recycled back into the mantle [1,2]. These types of parent materials are developed by dominant deposition processes, including erosion and sediment recycling, that store the geological characteristics of the preexisting rocks, such as the composition of rare earth elements (REEs) [3].

REEs are a group of fifteen transition metals from the lanthanide (Ln) series. In addition, scandium (Sc) and yttrium (Y) are usually included in REEs because of their similar chemical properties to the Ln family [[4], [5], [6], [7]]. REEs possess similar physical and chemical properties, having six electronic shell configurations and large ionic radii, and exist in the trivalent ionic form [8]. However, cerium (Ce) can additionally exist in the tetravalent form in oxidizing environments, while europium (Eu) may also occur in divalent species in reducing environments such as in seawater deposits [9]. REEs are grouped into light REEs (LREEs; Z = 57 to 63) and heavy REEs (HREEs; Z = 64 to 71) based on their atomic mass [2,10,11]. LREE concentrations are always greater than that of HREEs, and their natural abundance typically varies according to the Oddon-Harkin rule, in which REE concentrations decrease with the increasing atomic number [8].

The abundance of REEs in soils is highly influenced by the presence of parent materials [11,12]. Nevertheless, the favorable dissolution of primary minerals containing REEs from the parent materials, the complexation of REEs and their mobility in soil solution, the influence of soil colloids and secondary minerals, and the biological activity are the major controlling factors for the fractionation of LREEs and HREEs in the soils [13]. Many primary minerals indicate REE abundance differences that show enrichment or depletion of selected REEs or groupings of REEs; for instance, apatite is generally enriched with LREEs compared to zircon [12]. Consequently, the formation of clay particles due to the weathering of primary silicates acts as new carriers of REEs in soils and weathering profiles [8]. Moreover, the availability of Fe- and Mn-oxides in soils is known to contain REEs through coprecipitation, adsorption, surface complex formation, ion exchange, and penetration of the lattice [[8], [14]]. In fact, based on the previous studies, extractable crystalline and noncrystalline Fe oxides showed high affinity to the extractable REEs [5], elevated REE concentrations were observed in soils with high Fe oxide contents [12], and the retention of REE in the soil was increased by Fe oxide contents [15]. The formation of carbonates, phosphates, and organic matter (OM) also provide important functions in the characteristics of REEs in the soil [8,12,15,[14], [16]]. Furthermore, soil pH also contributes to the distribution of REEs through the fixation of REEs as an ionic complex adsorbed in the surfaces of the soil particles [8,17]. In acidic soil pH conditions, REEs are adsorbed as outer-sphere complexes in the basal surfaces, whereas REEs are adsorbed onto amphoteric sites at the edges of the clay particles at alkaline pH [1,8].

REEs are fractionated based on their atomic number and abundance, influenced by soil components, and enhanced by topographic position. Topography interacts with geological and geochemical processes, both indirectly (e.g., through alterations in soil properties) and directly (e.g., via erosion), shaping the spatial patterns of REE distribution [8,18]. These conditions promote the extensive breakdown of primary REE-bearing rocks, leading to reduced REE concentrations in the toposequence [19,20]. Furthermore, the increased weathering activity accelerates the release of REEs, which are subsequently transported downslope. Rainfall, often enhanced by mountain-induced orographic uplift or convective instability, plays a crucial role in redistributing REEs along altitudinal gradients [20,21]. Over time, the combination of high rainfall and greater weathering activity in elevated regions depletes REE concentrations there while contributing to their enrichment at lower elevations. Once released through weathering, REEs are mobilized and transported downslope, where they undergo fractionation based on ionic size, charge, and affinity for specific materials [8,22]. LREEs are more easily transported over longer distances because they are often incorporated into secondary REE-bearing phases, such as clays, allowing them to accumulate in depositional zones. In contrast, HREEs remain dissolved in soil solutions and are more prone to leaching, resulting in their relative depletion in lower landscape positions [23]. This size- and phase-specific behavior leads to higher concentrations of LREEs relative to HREEs in downslope and low-elevation areas [20]. In general, topography influences REE distribution through a combination of elevation-dependent weathering, downslope transport and fractionation, slope gradient effects on water movement, and aspect-driven microclimatic variations [18,20,21,24].

The behavior of REEs and other pedogenic factors in soils is highly regarded, yet most existing research focuses on their properties along vertical soil profiles [5,11,15,23], often neglecting the critical role of topographic conditions. Topography is a key determinant of soil formation and development, influencing the abundance, mobility, and bioavailability of REEs through its regulation of runoff, drainage, and erosion processes [24,25]. Variations in slope can significantly modify soil physical and chemical properties, such as pH, organic carbon content, and mineral composition, which in turn affect REE behavior and distribution [[26], [27], [28]]. Recognizing the need to address this gap, this study investigated the influence of pedogenic factors on the concentration, distribution, and fractionation of REEs in marine mudstone soils along a toposequence in Eastern Taiwan.

## 2 Materials and methods

### 2.1 Site description

**Fig. 1:**
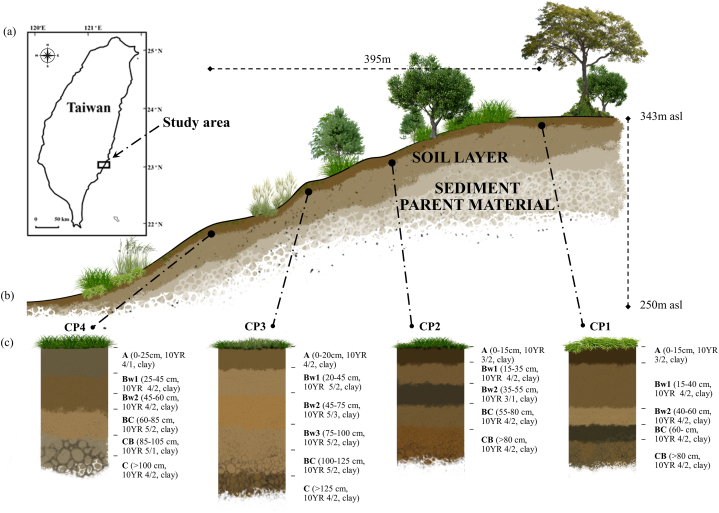
(a) Location of the study area, (b) studied pedons (CP1-summit, CP2-upper backslope, CP3-lower backslope, and CP4-footslope) along the sediment toposequence, and their (c) main morphological characteristics.

The study site was located in the ophiolite complex in Chishang, Taitung county, in which soil samples were mainly derived from mudstones in the eastern part of Taiwan (Fig. 1a). The geology of the study site is characterized by arc-continent collision [29], and the climate condition is characterized as humid and tropic with massive rainfall and tropical cyclones in summer. The average rainfall is 1800 mm, and the mean annual air temperature is 22.5 °C, which ranges from 18 °C to 27 °C. The dominant vegetation of the hill-shaped mountain is dominated by tropical deciduous trees (*Annonaceae* sp.), exotic trees (*Leucaena leucocephala*), and perennial grasses (*Oplismenus hirtellus*, *Carex obnupta*, *Cortaderia jubata*, *Saccharum spontaneum*).

Table 1 and Fig. 1b show the geographic information of the four studied pedons, comprising CP1, CP2, CP3, and CP4. The soil moisture and temperature regimes are udic and hyperthermic. The elevation of the study site ranges from 290 to 343 m above sea level with an increasing slope gradient (5–15 %) from the foot slope to the summit (Fig. 1b). All pedons were approximately south-facing, with pedon CP1 located on the summit, pedon CP2 on the upper backslope, pedon CP3 on the lower backslope, and pedon CP4 on the footslope. Based on the Soil Survey Staff [30], the four pedons were classified as Typic Dystrudepts.

**Table 1:** Geographic information and classification of the studied pedons.

Pedon	Location	Elevation	Slope	Landscape Position	Soil Classification^a^
		(m)	(%)		
CP1	N 23° 04′ 16.82″, E 121° 13′ 21.64”	343	15	Summit	Typic Dystrudept
CP2	N 23° 04′ 11.95″, E 121° 13′ 16.34”	322	15	Upper backslope	Typic Dystrudept
CP3	N 23° 04′ 10.05″, E 121° 13′ 14.72”	310	10	Lower backslope	Typic Dystrudept
CP4	N 23° 04′ 09.21″, E 121° 13′ 11.04”	290	5	Foot slope	Typic Dystrudept

a Soil Survey Staff [30].

### 2.2 Characterization of soil physical and chemical properties

Soil samples were collected from each horizon and placed in sealed and properly labeled containers. The soil samples were then air-dried and pulverized in the laboratory. The particle-size distribution of the soil samples was determined using the pipetted method [31], while bulk density (BD) was analyzed using the core method. Soil pH was measured using a mixture of soil and deionized water (1:1, w/v) with a glass electrode [32]. The Walkley-Black wet oxidation method was used to determine the soil organic carbon (OC) [33]. Cation exchange capacity (CEC) and base saturation (BS) percentage were determined by the ammonium acetate method (pH 7.0) [34]. Dithionite-citrate-bicarbonate (DCB) extraction was used for the determination of the dissolved crystalline and noncrystalline Fe- and Al-oxides [35] and was quantified using atomic absorption spectroscopy (AAS; PerkinElmer® AAnalyst 200, Waltham, USA).

Total major elements, including silicon (Si), aluminum (Al), iron (Fe), potassium (K), calcium (Ca), magnesium (Mg), sodium (Na), and manganese (Mn), were determined by wavelength dispersive X-ray fluorescence (WDXRF) spectrometer (Axios^mAX^ Advanced WDXRF, Malvern Panalytical, Malvern, UK). A mixture of HF-HNO_3_-HCl from the protocol recommended by the U.S. Environmental Protection Agency [36] was used for the digestion experiment for the determination of REEs. The digestion process was associated with a microwave system (Speedwave Entry, Berghof, Eningen, Germany) at a gradual increase of temperature of 180 °C in 10 min and further sustained at the same temperature and time. REEs in the digested solution were quantified using inductively coupled plasma mass spectrometry (ICP-MS; 7700e, Agilent, Santa Clara, CA, USA).

### 2.3 REEs normalization, fractionation indexes, and weathering proxy calculation

The concentration of individual REE in the soil samples was normalized with that of the upper continental crust (UCC) [2]. The fractionation between LREEs and HREEs was quantified using the fractionation proxies La_N_/Yb_N_, La_N_/Sm_N_, and Gd_N_/Yb_N_ ratios, where subscript N corresponds to the normalized values using UCC [37,38]. The anomalies (δ) of Ce [Ce_N_/(La_N_∗Pr_N_)^0.5^] and Eu [Eu_N_/(Sm_N_∗Gd_N_)^0.5^] were determined according to Compton et al. [39] since they exist in two oxidation states under general soil conditions and exhibit specific geochemical behaviors. The value above “1” (i.e., positive anomaly) suggests an enrichment, and a value below “1” (i.e., negative anomaly) indicates depletion in comparison with La and Pr (for δCe), and Sm and Gd (for δEu) [8].

The chemical index of alteration (CIA) was used as a proxy for the chemical weathering of the studied soil pedons. The CIA was calculated according to the equation by Nesbitt and Young [40] CIA = 100 x [Al_2_O_3_/(Al_2_O_3_ + CaO + Na_2_O + K_2_O)]. CIA increases with cation losses (Ca^2+^, K^+^, and Na^+^) when primary minerals (i.e., feldspars) transform into kaolinite during the soil weathering, and Al is considered relatively immobile in this alteration [41].

### 2.4 Quality assurance, quality control, and statistical analysis

A standard sample from REE-1 (Strange Lake deposit) from the Canadian Certified Reference Materials Project (CCRMP) was also digested and analyzed using the U.S. Environmental Protection Agency [36] sample digestion method. The recovery of the individual REE was as follows: La, 81.6 %; Ce, 85.1 %; Pr, 95.6 %; Nd, 99.0 %; Eu, 110.57 %; Gd, 111 %; Tb, 121 %; Dy, 112 %; Ho, 109 %; Er, 104 %; Tm, 107 %; Yb, 102 %; Lu, 98.5; Sc, 91.9 %, and Y, 72.7 %.

Statistical software R (version 4.1.0) [42] was used to perform Pearson's correlation coefficients (r) for the linear relationships among the soil properties and concentrations of major elements and REEs. The interrelationships among the variables and the relatedness among the studied pedons were determined by principal component analysis (PCA). The statistical significance levels were determined at the p-value of ∗*p* < 0.05, ∗∗*p* < 0.01 and ∗∗∗*p* < 0.001.

## 3 Results and discussion

### 3.1 Soil physical and chemical characteristics

Table 2 and Fig. 1c list the main morphological and the selected soil characteristics of the studied pedons from the marine sediments. No distinct variation was observed in the distribution of the soil particles in all studied pedons; however, the clay fraction (45.0 %–62.5 %) was much higher than the sand fraction, ranging from 2.5 % to 40.0 % in all pedons. The highest and lowest clay contents were observed in the lower backslope (CP3) and the foot slope (CP4), respectively. Generally, a lower soil bulk density was observed in the surface horizons in all soil pedons compared to that in the subsurface horizons. Soil pH values in all pedons were weak acidic (6.06–6.88), which could be considered a typical pH condition for developing soils in an uplifted marine terrace in eastern Taiwan [43]. Comparable moderate OC values (5.0–5.8 %) were observed in the surface horizons, while very low to low (1.3–3.6 %) values were found in the subsurface horizons [44]. The CEC values in the studied pedons were characterized as moderate, ranging from 14.5 to 27.3 cmol (+) kg^−1^. In general, the decreasing values of CEC relative to the increasing soil depth were probably influenced by the OC contents in the studied pedons, which can be observed in the decreasing OC values. The exchangeable Ca (3.62–10.6 cmol(+) kg^−1^) dominated the exchangeable cations in the studied soils, followed by Mg (0.45–1.12 cmol(+) kg^−1^). Usually, ophiolitic soils were characterized by the exchangeable Mg-enriched pattern, which was the inheritance from their serpentinite parent materials; however, the higher concentration of exchangeable Ca than that of Mg observed in this study indicated that soils in the studied ophiolite complex were mainly derived from sediments (Table 2) [[45], [46], [47]]. Percentage base saturation (BS) values (from 17.1 % to 82.2 %) in the studied soils ranged from low to high, where high values were observed in the C horizons in all pedons compared to the upper horizons, implicating the leaching of basic cations from the surface to the bottom of soil profiles [48].

**Table 2:** Selected properties of the studied pedons.

Exchangeable bases																
Pedon	Depth	Horizon	Sand	Clay	BD^a^	pH	OC^b^	CEC^c^	K	Na	Ca	Mg	BS^d^	Fe_d_^e^	Al_d_^e^	CIA^f^
	cm		%	%	g cm^−3^		%	cmol (+) kg^−1^					%			
CP1	0–25	A	17	50	1.4	6.26	4.97	27.3	0.20	0.02	3.91	0.60	17	78.4	10.7	77
	25–45	Bw1	12	55	1.4	6.53	2.87	23.5	0.11	0.01	3.70	0.74	19	87.8	10.9	78
	45–60	Bw2	10	55	1.5	6.75	2.54	20.6	0.11	0.01	4.10	0.82	24	78.4	9.97	78
	60–85	BC	12	52	1.6	6.63	2.08	19.7	0.11	0.04	3.93	0.85	25	76.1	8.75	72
	85–105	CB	30	45	1.6	6.78	1.86	16.4	0.12	0.09	6.62	0.90	47	75.9	7.30	77
	>105	C	18	50	nd	6.81	2.57	17.6	0.15	0.25	5.66	1.12	41	65.5	6.30	73
CP2	0–20	A	10	55	1.5	6.60	4.99	24.4	0.22	0.05	6.69	0.60	31	75.1	7.78	78
	20–45	Bw1	2	63	1.4	6.76	3.42	18.0	0.15	0.01	5.74	0.61	36	86.2	6.76	74
	45–75	Bw2	15	58	1.5	6.62	3.08	17.8	0.13	0.02	6.64	0.77	43	84.2	6.18	74
	75–100	Bw3	2	60	1.7	6.59	3.13	17.7	0.12	0.03	8.83	0.87	56	85.8	6.84	74
	100–125	BC	25	47	1.6	6.06	2.77	14.9	0.14	0.05	9.79	1.04	74	80.7	5.70	72
	>125	C	15	45	1.7	6.59	3.16	14.5	0.14	0.06	10.6	1.11	82	85.5	5.04	71
CP3	0–15	A	12	53	1.3	6.60	4.79	23.5	0.13	0.01	3.74	0.53	19	93.4	9.80	78
	15–35	Bw1	17	58	1.4	6.88	2.80	22.4	0.10	0.01	3.95	0.57	21	93.2	10.8	79
	35–55	Bw2	18	58	1.4	6.88	3.10	24.8	0.08	0.01	3.62	0.53	17	94.7	11.3	79
	55–80	BC	15	60	1.5	6.73	3.19	22.9	0.09	0.01	4.72	0.73	24	96.3	11.8	79
	>80	CB	28	58	1.4	6.68	2.77	20.5	0.10	0.05	5.28	0.68	30	91.9	10.1	79
CP4	0–15	A	38	45	1.2	6.29	5.43	24.2	0.13	0.01	4.41	0.45	21	77.1	8.60	76
	15–40	Bw1	30	50	1.6	6.54	2.95	20.3	0.17	0.02	5.29	0.54	30	85.6	9.81	77
	40–60	Bw2	40	45	1.4	6.28	2.89	20.3	0.08	0.02	4.23	0.61	24	85.9	9.40	77
	60–75	BC	25	55	1.5	6.64	2.92	20.4	0.07	0.02	4.47	0.69	26	87.4	9.54	77
	75–90	CB	33	50	1.3	6.77	1.31	20.5	0.07	0.02	4.31	0.62	24	85.7	9.84	77
	>90	C	30	48	1.4	6.73	1.93	19.5	0.07	0.03	4.61	0.64	27	86.0	10.1	77

a Bulk density.

b Organic carbon.

c Cation exchange capacity.

d Base saturation.

e DCB-extractable.

f Chemical index of alteration.

The DCB extractable Fe (Fe_d_) and Al (Al_d_) contents, which ranged from 65.5 to 96.3 g kg^−1^ and 5.04–11.8 g kg^−1^, respectively, varied and followed an inconsistent pattern as the soil depth increased in all the examined pedons. In general, noticeably elevated Fe_d_ concentrations were observed in B horizons compared to surface and C horizons, indicating the accumulation of free Fe in the developing mineral soil horizons. Based on the well-structured development in the mineral subsurface horizons (Bw) and the presence of low basic cations, the studied pedons were classified as Typic Dystrudepts [30]. The soil classification of the studied pedons was consistent with CIA values, which ranged from 71 to 79, representing moderately weathered [40].

### 3.2 Concentration and normalized pattern of rare earth elements in the soil pedons

The total concentration of REEs ranged from 95.7 to 230 mg kg^−1^, in which LREEs contributed 75 % of the total REEs with the concentration ranging from 57.0 to 177 mg kg^−1^ while HREEs contributed 25 % (ranging from 36.9 to 53.7 mg kg^−1^; Table 3). The highest ∑REE content was observed in CP4 (ranging from 176 to 221 mg kg^−1^), while pedon CP1 had the lowest concentration (ranging from 95.7 to 156 mg kg^−1^; Table 3). The results indicated that the distribution of soil in different landscapes contributed to the variations in REE levels by transportation to the downslope through erosion, resulting in elevated concentrations in CP4 and lower concentrations in CP1 [22]. Moreover, The REE concentrations observed in this study were comparable with those reported in previous research conducted in Taiwan. For example, soils derived from various parent materials, such as mudstone, sandstone, and shale, exhibited ∑REE concentrations ranging from 91.8 to 223.0 mg kg^−1^ [24]. However, these concentrations were approximately three times lower than those found in granite-derived soils in southeastern China [17]. This variation highlighted the significant influence of parent material and geographical region on REE levels in soils.

**Table 3:** Total concentrations of REEs (mg kg^−1^) in the studied pedons.

Depth	Horizon	LREEs						HREEs										∑REEs	∑LREEs	∑HREEs	∑LREEs/∑HREEs
(cm)		La	Ce	Pr	Nd	Sm	Eu	Gd	Tb	Dy	Ho	Er	Tm	Yb	Lu	Sc	Y				
Pedon CP1 (Summit)																					
0–25	A	24.7	18.3	6.90	27.2	5.38	1.47	7.03	0.81	3.89	0.76	2.11	0.34	2.13	0.35	10.4	14.3	126	84.0	42.1	2.0
25-45	Bw1	13.1	11.9	4.94	20.8	5.04	1.22	5.45	0.79	4.26	0.88	2.65	0.43	2.51	0.39	8.00	13.4	95.7	57.0	38.8	1.2
45-60	Bw2	32.9	22.6	8.38	32.0	6.29	1.69	8.22	0.86	3.99	0.84	2.33	0.34	2.09	0.36	11.2	16.7	151	104	46.9	2.2
60-85	BC	32.9	22.7	8.22	31.9	6.23	1.62	8.26	0.89	3.93	0.84	2.11	0.34	2.21	0.35	10.8	16.2	149	104	45.9	2.3
85-105	CB	29.5	20.7	7.52	28.8	5.78	1.48	7.32	0.75	3.39	0.63	1.98	0.27	1.92	0.30	7.60	13.2	131	93.7	37.4	2.5
>105	C	36.1	24.6	8.71	32.4	6.14	1.69	8.23	0.87	4.18	0.81	2.00	0.33	2.06	0.33	10.8	16.6	156	110	46.2	2.4
Pedon CP2 (Upper backslope)																					
0–20	A	35.8	25.0	9.11	34.6	6.98	1.84	7.92	0.88	4.25	0.83	2.23	0.36	2.28	0.36	12.0	15.5	183	137	46.6	2.9
20-45	Bw1	35.2	77.7	9.17	34.8	6.80	1.81	7.64	0.83	4.09	0.78	2.13	0.32	2.10	0.31	3.60	15.1	168	132	36.9	3.6
45-75	Bw2	38.6	82.5	9.50	35.6	6.94	1.90	9.56	0.94	4.44	0.91	2.37	0.32	2.34	0.34	11.6	19.5	230	177	52.3	3.4
75-100	Bw3	27.8	64.8	8.13	32.7	6.73	1.75	8.68	0.92	4.41	0.84	2.33	0.33	2.33	0.39	7.60	17.9	201	156	45.7	3.4
100-125	BC	38.4	80.3	9.40	35.9	7.15	1.99	7.36	0.39	5.59	1.20	3.24	0.51	3.17	0.48	12.4	19.4	189	136	53.7	2.5
>125	C	26.0	60.6	7.38	29.1	6.03	1.58	7.10	0.84	4.09	0.75	2.17	0.34	2.07	0.29	12.8	14.4	176	131	44.8	2.9
Pedon CP3 (Lower backslope)																					
0–15	A	26.5	63.5	7.88	30.6	6.46	1.66	9.04	0.93	4.39	0.87	2.48	0.38	2.35	0.36	10.8	18.3	163	113	49.9	2.3
15-35	Bw1	26.4	60.4	7.55	29.4	6.21	1.61	8.84	0.92	4.55	0.86	2.36	0.37	2.41	0.37	10.4	17.8	214	165	48.9	3.4
35-55	Bw2	39.1	83.1	9.76	36.4	7.01	1.82	9.00	0.97	4.39	0.88	2.31	0.34	2.43	0.35	6.40	18.2	220	175	45.2	3.9
55-80	BC	33.1	72.4	8.65	33.2	6.52	1.71	8.37	0.88	4.21	0.86	2.33	0.34	2.14	0.33	6.80	15.9	184	142	42.1	3.4
>80	CB	23.1	61.5	8.06	33.1	7.55	2.23	9.55	0.95	4.57	0.89	2.47	0.38	2.34	0.36	8.40	18.9	222	173	48.8	3.6
Pedon CP4 (Foot slope)																					
0–15	A	36.9	77.6	9.69	34.8	7.55	2.33	9.31	1.35	4.49	1.19	2.45	0.73	2.46	0.78	11.6	17.5	221	169	51.9	3.3
15–40	Bw1	35.7	74.9	8.90	32.0	6.79	2.13	8.65	1.29	4.05	1.10	2.35	0.67	2.33	0.67	12.0	16.4	210	160	49.5	3.2
40–60	Bw2	31.6	66.8	7.96	29.2	5.90	1.86	7.97	1.07	3.85	0.87	2.14	0.49	1.96	0.49	11.2	15.1	188	143	45.1	3.2
60–75	BC	36.7	77.9	9.01	33.9	6.79	2.01	8.84	1.14	4.22	0.94	2.25	0.51	2.21	0.48	9.60	16.6	213	166	46.8	3.6
75–90	CB	35.0	72.2	8.36	31.3	6.29	1.76	7.94	0.99	3.79	0.84	2.00	0.44	2.16	0.40	10.8	16.3	200	155	45.6	3.4
>90	C	30.3	62.8	7.40	27.6	5.45	1.44	7.07	0.83	3.33	0.72	1.82	0.37	1.81	0.35	10.8	13.6	176	135	40.7	3.3

In terms of ∑REE concentrations among studied pedons, considerably higher ∑REE concentrations were observed in the A horizons compared to Bw1, except in pedon CP2. The results showed an indication of the deposition of the soil particles containing REEs in the surface horizons, which might be influenced by higher OC contents in the A horizons (Table 2). OM has many negatively charged groups that provide a high capacity to complex, adsorb, or chelate positively charged REEs [8]. The lower concentrations of ∑REEs in Bw1 horizons showed an indication of leaching. Furthermore, Bw2, Bw3, BC, and CB horizons in all studied pedons tended to have higher ∑REE concentrations, which was similar to the elevated values of Fe_d_, indicating the influence of Fe oxides since they are known to be an important scavenger for REEs through coprecipitation, adsorption, surface complex formation, ion exchange, and lattice penetration [8,49].

The total concentration of individual REE showed variations relative to soil depth (Table 3). Ce exhibited the highest content among REEs, covering 31 % of the total REEs percentage, whereas Lu (0.22 %) was the lowest. In the natural environment, Ce concentration is always high because of the unique geochemical properties that can exist in multiple oxidation states (Ce^3+^ and Ce^4+^), which allows it to form stable complexes with a wide range of organic and inorganic compounds [50]. Furthermore, the individual REE concentrations in the studied soils derived from marine sediments were higher compared to those in the world-soil average and Earth's crust [51,52].

In general, the total REE concentrations in the studied marine sediment soils exhibited the following order: Ce > Nd > La > Y > Sc > Pr > Gd > Sm > Dy > Er > Yb > Eu > Tb > Ho > Tm > Lu; which is similar to the findings of Laveuf and Cornu [8] in which the content of REE decreases with the increasing atomic number based on the Oddo-Harkins rule. However, in terms of topographic positions, different REE series were observed: Nd > La > Ce > Y > Sc > Gd/Pr > Sm > Dy > Yb/Er > Eu > Tb/Ho > Tm/Lu (pedon CP1); Ce > Nd > La > Y > Sc > Gd/Pr > Sm > Dy > Yb/Er > Eu > Tb/Ho > Tm/Lu (pedon CP2 and CP3); Ce > La > Nd > Y > Sc > Gd/Pr > Sm > Dy > Yb/Er > Eu > Tb/Ho > Tm/Lu (CP4), with few exceptions. The studied sediment soils showed enrichment of Gd in pedon CP1 to CP4, elevated Nd levels in pedon CP1, a depletion of Sm concentration from pedon CP1 to CP4, depleted Ce values in pedon CP1, and decreased Nd content in pedon CP4. All soil samples showed La, Ce, and Nd were the most abundant, while Tb and Lu had the lowest concentrations in the studied sediment soils in the ophiolite complex. La, Ce, and Nd are the common REEs that have higher concentrations, and Lu has the least compared to the group of 17 transient metals [9,53].

**Fig. 2:**
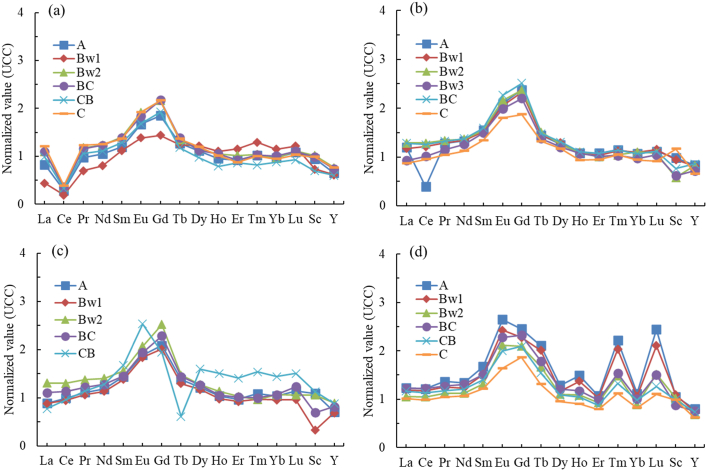
UCC-Normalized REE patterns of soil horizon samples in all pedons: (a) pedon CP1 (summit); (b) pedon CP2 (upper backslope); (c) pedon CP3 (lower backslope); and (d) pedon CP4 (foot slope).

The concentrations of individual REE in the soil samples were normalized through UCC, providing their depletion or enrichment information during the soil formation processes (Fig. 2). The pronounced drop of Ce indicated the depletion in the gradual rising trend of LREEs in pedon CP1, resulting in negative anomaly values and influencing the status of LREEs (Fig. 2a; Table 4). The negative Ce anomaly in pedon CP1 was a succession effect in the previous reducing marine conditions, in which the Ce desorbed from particulate matter, leading to considerable Ce depletion with respect to its REE neighbors [50]. Except on the Ce in the surface horizon of pedon CP2, a clear LREEs enrichment was observed in pedons CP2, CP3, and CP4, reflecting a flat and gradually rising pattern relative to the increasing atomic number from La to Eu (Fig. 2b, c, and 2d). The enrichments of LREEs in CP2, CP3, and CP4 corroborated the elevated concentrations of Fe_d_ (Table 2). Furthermore, slight HREE enrichments are observed in pedons CP1, CP2, and CP3, which can be generally noticed in the gradual decreasing patterns. Pedon CP4 showed an incomparable HREEs spider pattern, with decreasing values from Gd to Dy and higher values in Ho, Tm, and Lu (Fig. 2d). The higher values in most of the HREEs in pedon CP4 expressed the enrichments, which were pronounced in A and Bw1 horizons, indicating the deposition of particulate matters containing HREEs. Considering the location of the studied marine soils, the depletion of Ce might be affected by the constant movement of precipitation and erosion.

**Table 4:** Fractionation of UCC-normalized REEs and Ce and Eu anomalies of the studied pedons.

Depth cm	Horizon	La_N_/Yb_N_	La_N_/Sm_N_	Gd_N_/Yb_N_	δCe	δEu
Pedon CP1 (Summit)						
0-25	A	0.9	0.7	1.9	0.3	1.1
25-45	Bw1	0.4	0.4	1.3	0.3	1.1
45-60	Bw2	1.2	0.8	2.3	0.3	1.1
60-85	BC	1.1	0.8	2.2	0.3	1.1
85-105	CB	1.1	0.8	2.2	0.3	1.1
>105	C	1.3	0.9	2.3	0.3	1.1
Pedon CP2 (Upper backslope)						
0-20	A	1.1	0.8	2.2	0.3	1.1
20-45	Bw1	1.1	0.9	2.1	1.0	1.1
45-75	Bw2	1.2	0.8	2.1	1.0	1.1
75-100	Bw3	1.0	0.6	2.3	1.0	1.1
100-125	BC	1.2	0.8	2.4	1.0	1.1
>125	C	0.9	0.7	2.0	1.0	1.1
Pedon CP3 (Lower backslope)						
0–15	A	0.9	0.6	2.0	1.0	1.1
15-35	Bw1	0.9	0.6	2.1	1.0	1.1
35-55	Bw2	1.2	0.8	2.4	1.0	1.1
55-80	BC	1.0	0.8	2.2	1.0	1.1
>80	CB	0.5	0.5	1.4	1.0	1.4
Pedon CP4 (Foot slope)						
0–15	A	1.1	0.7	2.2	0.9	1.3
15–40	Bw1	1.1	0.8	2.2	1.0	1.3
40–60	Bw2	1.2	0.8	2.4	1.0	1.2
60–75	BC	1.2	0.8	2.3	1.0	1.2
75–90	CB	1.2	0.8	2.1	1.0	1.2
>90	C	1.2	0.8	2.3	1.0	1.1

### 3.3 Fractionation of soil REEs and anomalies of Ce and Eu

The topographic position of the studied soil was important for the distribution of REEs and influencing the fractionation of LREEs over HREEs in the soil profiles. Higher average ∑LREEs/∑HREEs ratios were observed in pedons CP2, CP3, and CP4 with average values of 3.2, 3.1, and 3.3, respectively, relative to pedon CP1 (2.1) (Table 3). The average ratio in pedon CP1 was comparable to the UCC value (2.8), while the other pedons contained higher values. The observed REE fractionation across the studied pedons can be attributed to underlying geochemical mechanisms governing their mobility and retention in soils [8,13,18]. HREEs exhibit greater solubility and a weaker affinity for adsorption onto secondary mineral phases such as clays and Fe/Al oxides, making them more mobile and susceptible to leaching [23]. Conversely, LREEs are preferentially adsorbed onto these mineral surfaces and retained in soils, especially in depositional zones. These retention processes are enhanced in lower landscape positions, where conditions such as prolonged water residence time and reduced erosion favor the accumulation of LREEs [18,23,24]. The progressive enrichment of LREEs at lower elevations is also influenced by the translocation and redistribution of REEs through erosion and meteoric water during pedogenesis [24]. The average La_N_/Yb_N_ ratios in the profiles reflect this fractionation, with depletion in pedons CP1 (0.98) and CP3 (0.92) and enrichment in pedons CP2 (1.07) and CP4 (1.17) (Table 4). Additionally, higher La_N_/Sm_N_ (0.73–0.83) and Gd_N_/Yb_N_ (2.19–2.27) ratios in pedon CP4 indicate substantial accumulation of LREEs in soils at lower elevations.

A noticeable trend of increasing δCe values was observed in the studied soils, ranging from 0.3 to 1.0 (Table 4). Pronounced negative δCe values were observed in pedon CP1, with an average value of 0.3, while comparable no anomalies were observed in pedons CP2, CP3, and CP4 (Table 4; Fig. 2). The negative δCe values in the studied soils were strongly associated with the parent materials developed from the previous anoxic or marine environments (e.g., 11,49,55). According to Zhang and Shields [50], marine sediments evidently showed negative δCe, which might inherited directly from the authigenic phase. Furthermore, the relative immobility of REEs from their origin leads to low concentrations of the elements in the water column of the marine environment, influencing the magnitude of Ce anomaly (negative) in the forming parent materials (marine sediments). The immobility of Ce from the previous environment is influenced by the adsorption of all REEs onto reactive particle surfaces (e.g., Fe/Mn oxyhydroxides), in which reactive oxides serve as electron acceptors [50]. In addition, landscape positions also greatly influenced the run-off and the development of the soils. In most cases, the development of soils in higher elevated areas often accompanied by intensive leaching and erosion [54], leading to negative δCe in the soils from the summit and positive δCe in the footslope. In this study, no anomalies of Ce were found in the soils from the lower positions of the landscape; however, the differences in Ce anomalies in the studied pedons evidently showed the influence of the landscape positions.

The positive δEu, ranging from 1.1 to 1.4 (Table 4; Fig. 2), was observed in all studied profiles, which was the influence of their parent material. The marine sediment parent material typically showed positive δEu, which was attributed to the reducing conditions of the previous environment and the detrital material signature from the weathering of source rocks [10,55,56,57]. In the studied pedons, the soils that developed in the lowest part (pedon CP4) of the landscape showed higher positive δEu values (average value of 1.2) compared to the soils from pedons CP1, CP2 and CP3, indicating the solubility and mobility of the Eu from the upper slope down to the lower slope. Comparable δEu values were observed within the studied pedons.

The fractionation and mobility of REEs across landscape positions have important environmental implications, particularly in altering soil geochemistry and elemental cycling [8,18]. The enrichment of LREEs in lower-elevation soils highlights their role as depositional zones, potentially affecting nutrient availability and microbial activity [24]. Negative δCe values in higher-elevation soils reflect oxidative conditions and parent material influences, while positive δEu values suggest enhanced mobility and redistribution of Eu downslope, contributing to localized elemental imbalances [10,55,57]. These processes emphasize the role of topography in shaping REE dynamics, which can impact ecosystem stability, vegetation patterns, and nutrient cycling. Understanding these dynamics is crucial for sustainable land management and assessing potential risks of REE contamination in water systems, especially in erosion-prone or anthropogenically disturbed landscapes.

### 3.4 Correlations between soil properties, fractionation proxies, and REE

Further analysis of the geochemical behavior of REEs was determined using Pearson's correlation analysis to quantify the relationships among soil basic properties, selected major elements, fractionation indices, anomalies, weathering index, and REEs (Table 5). No significant correlation was found for soil pH with the ∑REEs, REEs subgroups, and theirfractionations (∑LREEs/∑HREEs) (Table 5). The result contrasted with the typical trend between REEs and soil pH because the hydrolytic stability and activity of REEs increase relative to increasing pH values, and LREEs exhibit greater hydrolysis strength than HREEs [58]. With the decreasing elevations, the REE concentrations were distinctively increased (ranging from 95.7 to 230 mg kg^−1^), but the values of the soil pH remained comparable (Table 2; Table 3). Weak acidic pH conditions in developing soils promoted the solubility of the REEs by the presence of protons that react with the metal-containing minerals, resulting in the release of REEs into the soil solution [59], which further led to the accumulation of REEs in the soils on the lower slope (i.e., pedon CP4; Table 3).

**Table 5:** Pearson's correlation matrix among selected soil properties, weathering index, and REE proxies in the studied soil samples (n = 24). OC: organic carbon; CEC: cation exchange capacity; CIA: chemical weathering of alteration; δCe: Ce anomaly; δEu: Eu anomaly.

	Sand	Clay	pH	OC	Fed	CIA	∑REEs	∑LREEs/∑HREEs	La_N_/Yb_N_	La_N_/Sm_N_	Gd_N_/Yb_N_	δCe	δEu
Sand													
Clay	−0.69∗∗∗												
pH	−0.3	0.47∗											
OC	−0.18	0.04	−0.43∗										
Fed	−0.04	0.45∗	0.21	−0.04									
CIA	0.2	0.26	0.29	0.13	0.48∗								
∑REEs	0.23	0.18	−0.11	0.17	0.33	−0.05							
∑LREEs/∑HREEs	0.18	0.19	−0.05	0.1	0.35	−0.08	1.00∗∗∗						
La_N_/Yb_N_	0.24	−0.24	0.08	−0.2	−0.35	−0.24	0.41	0.47∗					
La_N_/Sm_N_	0.22	−0.22	0.09	−0.2	−0.37	−0.22	0.36	0.42∗	1.0∗∗∗				
Gd_N_/Yb_N_	0.1	−0.18	0.02	−0.07	−0.27	−0.22	0.39	0.46∗	0.94∗∗∗	0.87∗∗∗			
δCe	0.24	0.14	−0.03	−0.01	0.70∗∗∗	0.02	0.77∗∗∗	0.80∗∗∗	0.08	0.01	0.11		
δEu	0.63∗∗	−0.25	−0.34	0.11	0.06	0.12	0.41	0.30	−0.16	−0.18	−0.26	0.37	

∗, ∗∗, and ∗∗∗ Significant at *p* < 0.05, 0.01, and 0.001.

The abundant negatively charged groups of OM also provide high complex capacity, adsorption, and chelation to the positively charged REEs [[8], [14]]. Organic matters are preferred to complex with HREEs rather than LREE in soils with near-neutral pH [60,61]. In contrast, the study of Feitosa et al. [11] showed no relationship between total HREEs and OC, while LREE is highly associated with OC. However, in this study, no correlation was observed among OC, ∑REEs, REEs subgroups, and REEs fractionation proxies. Very small variations of OC values were observed among pedons in this study, and generally, subsurface horizons contained elevated REE concentrations where OC was low (Table 2; Table 3). The result implied that OM was only a scavenger for REEs since the complexation varies strongly based on the type, OM composition and content, soil pH, and redox condition (Table 5) [8].

In the soil environment, the high surface area of clay particles provides greater adsorption sites for elements, including REEs [62]. However, REEs have relatively larger ionic radii, thus more diffuse electron clouds, and are generally less attracted to the soil particles [63], which reflects no correlation result between REEs and clay particles was found in this study (Table 5). Furthermore, low clay percentages (45–55 %) were observed in pedon CP4, where REE concentrations were considered enriched (Table 2; Table 3). No correlations were found between the Fe_d_ and the REEs in the studied soils from marine sediments; however, Fe_d_ and REEs concentrations tended to increase together relative to the decreasing elevations (Table 2; Table 3; Table 5). The result indicated that Fe-oxides influenced the redox potential of REEs and acted to scavenge in soils through one or a combination of coprecipitation, adsorption, surface complex formation, ion exchange, and penetration of the lattice [8,[12], [14]] that showed the positive correlations (*p* < 0.001) with the δCe (Table 5). Under oxidizing conditions, especially in well-drained soils, Fe-oxides act as oxidizing agents of Ce(III) to form Ce(IV), and the latter species are more stable and form solid phases, immobilized and precipitate [9]. The statistical analyses also showed high positive correlations (*p* < 0.001) between ∑REEs and ∑LREEs/∑HREEs, reflecting the enrichment of LREEs over HREEs. The behavior of REEs in the natural environment in relation to the other components of soil indicated that elements preferred to occur as group-free species and had no direct associations in the developing soils (Inceptisols). REEs occur in the environment as a group due to their similar chemical and physical properties [12].

**Fig. 3:**
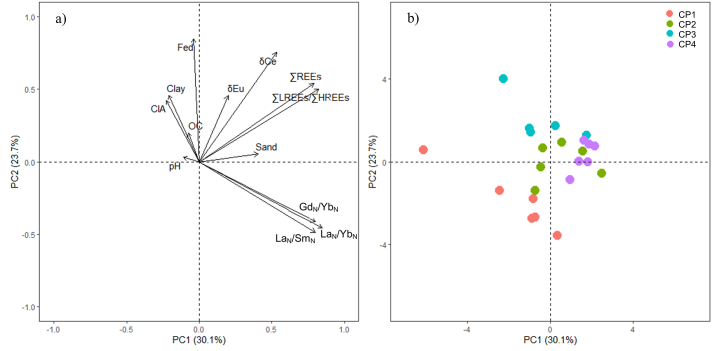
Loading plot (a) and score plot (b) for the PCA of soil properties, weathering indices, and REE proxies in the studied soil samples (n = 21). OC: organic carbon; CEC: cation exchange capacity; CIA: chemical index of alteration; δCe: Ce anomaly; δEu: Eu anomaly.

For further understanding of the relationships between basic soil components and REEs, the variables in Pearson's correlation analysis were further used in PCA, and the five principal components are listed in Table 6. Cartesian coordinates of PC1(30.1) and PC2 (23.7) were used to illustrate better the interactions among soil sample variables (n = 23). The selected basic soil properties and weathering index were weakly loaded in PC1 (−0.11 to 0.21) (Table 6). PC2 showed that the effect of the soil basic properties (clay, pH, OC, Fe_d_), except sand particles, was only marginal on the total REEs, REEs subgroups, and REEs fractionation proxies (Fig. 3a). In general, the content of REEs in soils was highly controlled by their parent materials, in which sediment parent materials typically have greater REE concentrations [8,12,23]. Furthermore, marine sediment parent materials are considered the source and sink of trace metals, and the sand particles that formed during the soil formation are typically identified as primary minerals that have not experienced significant weathering [1,2]. These close relationships of parent materials to the concentrations and distributions of REEs in the soil were substantiated by the interactions of sand particles to the REEs in PC1. According to Laveuf and Cornu [8] and Lee et al. [64], marine sediments typically presented positive δEu because of the feldspars it contains, and during the weathering condition, Eu would be released in a reduced form will be incorporated into the solid fractions, both detrital and weathering products. PC2 showed positive interactions between Fe_d_ and REEs, especially with δCe, indicating the strong influence of Fe oxides on the redox conditions of REEs in the soil environment. Furthermore, compared with soil properties that positively and majorly loaded in PC2, the moderate loadings of REE fractionation proxies in PC1 represented the negligible impact of these soil properties on the groupings of LREEs and HREEs during the soil formation; hence, REEs preferred to occur as a group in the environment due to similar chemical and physical properties [12]. The clustering of soil samples was shown in the score plot, wherein the pedon CP1 was separated from the overlapping samples in pedons CP2, CP3, and CP4 (Fig. 3b). However, clusters were not observed in the distinguished prevalence of total REEs, LREEs, HREEs and REEs fractionation proxies, demonstrating that the soil basic properties do not directly control the behavior of REEs in the soils derived from marine sediments (Fig. 3b).

**Table 6:** Results of PCA for selected soil properties, weathering indexes, and REE proxies in the studied soil samples (n = 21).

	PC1	PC2	PC3	PC4	PC5
Sand	0.21	0.03	−0.46	0.44	−0.12
Clay	−0.11	0.26	0.48	−0.16	−0.04
pH	−0.06	0.02	0.47	0.42	−0.01
OC^a^	−0.04	0.11	−0.15	−0.60	−0.56
Fe_d_^b^	−0.02	0.48	0.17	0.17	0.01
CIA^c^	−0.11	0.24	0.05	0.38	−0.73
∑REEs	0.40	0.31	0.02	−0.16	0.01
∑LREEs/∑HREEs	0.42	0.28	0.08	−0.14	0.06
La_N_/Yb_N_	0.43	−0.26	0.13	0.07	−0.13
La_N_/Sm_N_	0.40	−0.28	0.14	0.08	−0.14
Gd_N_/Yb_N_	0.40	−0.24	0.17	−0.05	−0.18
δCe^d^	0.27	0.43	0.01	0.02	0.25
δEu^e^	0.10	0.26	−0.45	0.11	0.04
eigenvalue	3.91	3.09	2.38	1.46	1.00
% of variation	30.11	23.73	18.30	11.24	7.67
% cumulative variance	30.11	53.84	72.13	83.38	91.05

a Organic carbon.

b DCB-extractable.

c Chemical index of alteration.

d Ce anomaly.

e Eu anomaly.

## 4 Summary and conclusions

The soils derived from marine sediments along the toposequence were dominated by LREEs, with the highest concentrations and fractionation observed in the footslope (CP4) and the lowest in the summit. Landscape position influenced Ce and Eu anomalies, with positive δEu and no δCe values in lower landscape positions linked to erosion and leaching. REE geochemical behavior was not significantly controlled by soil pH, OC, clay, or Fe oxides;however, δCe showed a strong positive correlation with Fe_d_, indicating Fe-oxides' impact on REE redox potential. A positive correlation between sand particles and δEu suggested that parent material controlled REE concentrations. The high correlation between ∑REEs and ∑LREEs/∑HREEs indicated LREE enrichment, as HREEs are more mobile. Overall, the concentration, distribution, and fractionation of REEs suggest they behave as distinct elements within the developing Inceptisols, influenced by their similar chemical and physical properties. Further investigation of various soil types is necessary to corroborate our conclusions regarding REE fractionation and its implications for the environmental effects of critical technology development.

## CRediT authorship contribution statement

**Marvin D. Cascante:** Writing – original draft, Visualization, Investigation, Data curation, Conceptualization. **Cho-Yin Wu:** Writing – review & editing, Validation. **Zeng-Yei Hseu:** Writing – review & editing, Validation, Resources, Funding acquisition, Conceptualization.

## Data and code availability statement

Data will be made available on request. For requesting data, please write to the corresponding author.

## Ethical clearance

No ethical clearance to be declared.

## Declaration of competing interest

The authors declare that they have no known competing financial interests or personal relationships that could have appeared to influence the work reported in this paper.
